# Repeated oral esketamine in patients with treatment resistant depression and comorbid posttraumatic stress disorder

**DOI:** 10.1016/j.heliyon.2023.e15883

**Published:** 2023-05-04

**Authors:** J.K.E. Veraart, M. van Westenbrugge, J.E. van Wulfften Palthe, A. van der Meij, R.A. Schoevers, J. de Jong

**Affiliations:** a PsyQ Depression Treatment Centre, Parnassia Psychiatric Institute, The Hague, the Netherlands; b Department of Psychiatry, University of Groningen, University Medical Centre Groningen, Groningen, the Netherlands; c Department of Psychiatry, Erasmus Medical Centre Rotterdam, Rotterdam, the Netherlands; d Pro Persona Mental Health Care, Depression Expertise Centre, Nijmegen, the Netherlands; e PsyQ Psychotrauma Department, The Hague, the Netherlands

**Keywords:** Treatment resistant depression (TRD), Posttraumatic stress disorder (PTSD), Esketamine

## Abstract

**Introduction:**

Ketamine and its S-enantiomer esketamine are novel pharmacotherapeutic options for treatment resistant depression (TRD). There is growing evidence on the efficacy for other psychiatric disorders, including posttraumatic stress disorder (PTSD). It is hypothesized that psychotherapy may further potentiate the effects of (es)ketamine in psychiatric disorders.

**Methods:**

Repeated oral esketamine was prescribed once or twice weekly in five patients suffering from TRD and comorbid PTSD. We describe the clinical effects of esketamine and report data from psychometric instruments and patients’ perspectives.

**Results:**

Esketamine treatment duration ranged from six weeks to a year. In four patients, we observed improvement in depressive symptoms, increased resilience and more receptiveness to psychotherapy. One patient experienced symptom worsening in response to a threatening situation during esketamine treatment, highlighting the need for a safe setting.

**Discussion:**

(Es)ketamine treatment within a psychotherapeutic framework appears promising in patients with treatment resistant symptoms of depression and PTSD. Controlled trials are warranted to validate these results and to elucidate the optimal treatment methods.

## Highlights

• (Es)ketamine is thought to potentiate psychotherapeutic interventions in patients with psychiatric disorders.
• Repeated oral esketamine seemed to improve resilience and receptiveness to therapy in patients with TRD and comorbid PTSD.
• Elaborate preparation, holding and a safe environment were found to be of utmost importance during esketamine treatment.

## 1 Introduction

Co-occurrence of major depressive disorder (MDD) and PTSD is common [1], and is associated with a more severe clinical presentation and reduced treatment efficacy than either disorder alone [[2], [3], [4]]. Serotonin reuptake inhibitors, recommended as first-line treatment for MDD and PTSD, have shown limited efficacy in both disorders [5,6]. For the past two decades, the rapid and robust antidepressant effects of the N-methyl-d-aspartate (NMDA) receptor antagonist ketamine and its S-enantiomer in patients with treatment resistant depression (TRD) have gained interest [[7], [8], [9]]. Moreover, ketamine is increasingly being studied for its potential in other psychiatric disorders, including posttraumatic stress disorder (PTSD) [[10], [11], [12], [13], [14], [15]]. Evidence for ketamine as a new pharmacotherapeutic intervention for PTSD has emerged from several case reports [[16], [17], [18]], retrospective and open label trials [19,20] and randomized controlled trials (RCTs). In 5 RCTs, single as well as repeated intravenous (IV) ketamine infusions of 0.5 mg/kg have shown rapid clinical improvement and good tolerability in patients with PTSD [21]. The most recent RCT showed no significant difference in PTSD symptom improvement between the ketamine and control groups in veterans and active duty military [22]. This was attributed to high response in the control group, resulting in small differences between the groups, an effect that is common in this field [23]. Clinical trials of PTSD show placebo response rates ranging from 19 to 62% [[24], [25], [26]]. On the other hand, clinical effect sizes suggest particularly poorer treatment response among veterans and military personnel compared to other PTSD populations [27].

In addition, ketamine may potentiate psychotherapeutic interventions in patients with different psychiatric disorders [[28], [29], [30]]. In this regard, a single IV infusion of ketamine as augmentation to trauma-focused psychotherapy has been proposed by Veen et al. [31]. The observational case series of Keizer et al. [32] described patients with chronic neuropathic pain and PTSD receiving ketamine infusions (dose ranging from 2 μg/kg/min to 15 μg/kg/min) and psychotherapy. Ketamine induced breakthroughs in focusing on trauma memories. In the placebo controlled cross-over study of Pradhan et al. [33], ketamine infusion was combined with mindfulness-based extinction and reconsolidation psychotherapy in patients with chronic PTSD. One IV ketamine infusion of 0.5 mg/kg was preceded by the controlled activation of traumatic memories and followed by mindfulness exercises. A more durable reduction in PTSD symptoms was achieved in the ketamine group when compared to the placebo group.

It remains to be investigated whether oral esketamine could be a promising candidate for combined pharmaco- and psychotherapy in patients with TRD and PTSD. In this case series, we report on the effects of repeated oral esketamine in patients who showed difficulties in initiating or tolerating trauma-focused treatment, specifically looking at the potential of repeated oral esketamine in enabling psychotherapy.

## 2 Methods

Combined treatment consisting of oral esketamine plus psychological treatment was offered to patients with TRD and comorbid PTSD in a specialized inpatient treatment centre for patients with depression. The participants were treated according to an off-label ‘compassionate use’ treatment protocol that was passed by the Medical Ethics Committee of the University Medical Center Groningen as not subject to the Netherlands act on medical research involving human subjects. Patients gave written informed consent for off-label treatment with generic oral esketamine (a liquid formulation) twice weekly. Doses started at 0.5 or 1 mg/kg and were individually titrated up to a maximum of 3 mg/kg, based on antidepressant effects and tolerability of the acute effects. Psychiatric side effects such as anxiety or flashbacks were managed by providing regular check-ins, guidance, music, breathing exercises and basic supportive touch such as handholding (all based on patient preferences). Depression severity change was assessed by the Hamilton Depression Rating Scale (HDRS) [34] and Inventory of Depressive Symptomatology – Self Rated (IDS-SR) [35] at baseline and after 6 weeks of treatment. In addition, patient perspectives were collected by the treating psychiatrist by asking the patients to write down a few lines about their experiences with the esketamine treatment. Each case will be described by first providing information on the psychiatric and family history, followed by the course of esketamine treatment and its effects, and the patient account.

## 3 Results

### 3.1 Case 1

A 65-year-old patient diagnosed with severe and chronic TRD and complex PTSD presented with low mood, emotional lability, anhedonia, loss of energy, intrusions and flashbacks, hypervigilance, suicidal thoughts and a wish for euthanasia. The traumatic experiences consisted of maltreatment and emotional neglect in her early childhood and repeated sexual abuse. Recurrent depressive episodes started at the age of 42 years. Current medication consisted of fluoxetine, bupropion, trazodone, lorazepam and oxycodone (for chronic pain). There was a family history of depression, borderline personality disorder and autism spectrum disorder. Previous pharmacotherapy consisted of treatment with selective serotonin and noradrenaline reuptake inhibitors (SSRIs and SNRIs), mirtazapine, bupropion, tricyclic antidepressants (TCAs), lithium, monoamine oxidase inhibitors (MAOIs), pregabaline, quetiapine, doxazosine and benzodiazepines. Trauma therapy including Eye Movement Desensitization and Reprocessing (EMDR) had been unsuccessful because the patient felt flooded with anxiety and it triggered self-harm and suicidality. Esketamine was titrated to a maximum dose of 2.0 mg/kg. Acute side effects consisted of drowsiness, light-headedness, headache, numbness of the body, nausea, and dizziness. Intrusions and dissociation occurred and resulted in anxiety. The patient repeatedly experienced flashbacks of the traumatic events, occurring within 24 h after esketamine administration. The staff offered reassurance and holding. The patient experienced a reduction in the burden caused by the traumatic experiences after experiencing flashbacks. Her mood improved, she was less emotionally unstable and more motivated to undertake activities. The total HDRS and IDS-SR scores decreased from baseline to week 6 from 29 to 25 and 56 to 47 respectively. Trauma therapy was then retried in the form of imaginary exposure (IE), thrice weekly on days that the patient did not receive esketamine. We observed a broader ‘window of tolerance’ during exposure in comparison to the earlier trial of trauma therapy. After several weeks of combined IE and esketamine treatment, flashbacks and intrusions had become less frequent. The esketamine treatment had a total duration of five months and was tapered down before discharge from the ward. We intended to continue outpatient psychotherapy, but this was interrupted due to COVID-19 restrictions. The patient reported that for the first time, she felt safe and resilient enough to talk about her traumatic experiences. The complete narrative can be found in Table 1.

**Table 1:** Patient characteristics, esketamine treatment, depressive symptoms and patient's narratives.

	Patient characteristics	Esketamine treatment	HDRS & IDS-SR	Patient's perspective
Case 1	Female 65 yo TRD, PTSD	Titrated to 2 mg/kg, twice weekly for 5 months	HDRS 6 → 29 IDS-SR 56 → 47	“The combined treatment with esketamine and IE was arduous because the flashbacks and the exposure were difficult to endure. However, the esketamine treatment enabled me to carry on during psychotherapy. It felt calmer in my mind and less chaotic, and it was ‘easier to let the misery come out’. I had never wanted to talk about the traumatic experiences but now felt safe and resilient enough to do so.”
Case 2	Female 66 yo TRD, PTSD	Titrated to 2 mg/kg, once or twice weekly for 5 months	HDRS 18 → 8 IDS-SR 35 → 45	Missing.
Case 3	Female 57 yo TRD, PTSD	Titrated to 0.75 mg/kg, twice weekly for 6 weeks	HDRS 22 → 26 IDS-SR 58 → 32	“I had put all my hopes on esketamine as the results are very promising. The first session was a strange experience, I couldn't see anymore, couldn't speak properly and I couldn't read well. I was also very nauseous. With the higher dose, I didn't respond well. My whole body cramped, and I couldn't move anymore. Everything hurt from head to toe. I had a very bad feeling in my head, that the trauma was coming back. The last session was like a nightmare from the past, the cramping of my body and the feeling that the trauma had taken over.”
Case 4	Female 34 yo TRD, PTSD, personality disorder, OCD and binge-eating	Titrated to 1.5 mg/kg, twice weekly, treatment ongoing (>6 weeks)	HDRS 30 → 11 IDS-SR 57 → 27	“The esketamine treatment has clearly improved my depression. When I'm depressed, my thoughts are running from left to right, everything is mixed up and I become entangled in it. During the esketamine I feel disconnected from reality. Thoughts, but also (traumatic) events and (unprocessed) emotions come and go. I've noticed that at that time, I can better let go and observe, and put things into perspective much better. After the esketamine dose, I feel significantly better within a few hours. It seems to help me feel better about myself and to feel calmer.”
Case 5	Female, 54 yo TRD, PTSD	Titrated to 3 mg/kg, twice weekly, treatment ongoing (> a year)	HDRS 25 → 15 IDS-SR missing	Not requested.

HDRS = Hamilton Depression Rating Scale, IDS-SR = Inventory of Depressive Symptomatology – Self Rated, OCD = obsessive compulsive disorder, PTSD = posttraumatic stress disorder, TRD = treatment-resistant depression.

### 3.2 Case 2

The second case concerns a 66-year-old patient with severe and chronic TRD and PTSD. She presented with a low mood, anxiety, loss of energy, and low self-esteem. In her childhood, she had been subjected to affective neglect and repeated sexual abuse. She had a family history of MDD. At admission, she was treated with clomipramine, mirtazapine, and diazepam. Previous pharmacotherapy consisted of SSRIs, mirtazapine, lithium augmentation and a single dose of psilocybin (dose unknown) through an experimental trial. She had received several forms of psychotherapy including EMDR, which had not been successful because the patient rationalized events and avoided emotional involvement. Esketamine was titrated to a maximum dose of 2.0 mg/kg. Acute side effects consisted of blurred vision, numbness of the face and dizziness. The patient experienced flashbacks of her traumatized youth after esketamine ingestion. These flashbacks lasted for about 30–60 min and the staff reassured the patient verbally and by holding her hand. Furthermore, she experienced nightmares about her traumatic experiences. The patient assumed the nightmares had been present before esketamine treatment, causing distress in the morning, but now she had become more aware of them. Furthermore, she reported the emotional load to be reduced. Depressive symptoms as assessed by the HDRS decreased from 18 to 8 whereas the IDS-SR increased from 35 to 45. EMDR was re-initiated with positive results. After 5 months of oral esketamine administration once or twice a week, combined with EMDR in the last weeks, the patient was discharged. Oral esketamine was terminated while the patient continued trauma focussed psychotherapy in an outpatient setting. She did not provide a description of her own experience with esketamine treatment.

### 3.3 Case 3

The third case was a 57-year-old patient with TRD suffering from low mood, anxiety, hypervigilance, nightmares and insomnia, loss of concentration and suicidal ideation. Two years prior to admission she had witnessed a deadly attack, after which she suffered from PTSD. She had received EMDR with good result, but the PTSD symptoms re-emerged, leading to the current depressive episode. Current pharmacotherapy consisted of sertraline, lorazepam, doxazosine and trazodone. Previous pharmacotherapy had consisted of citalopram, venlafaxine, nortriptyline, lithium augmentation and tranylcypromine. Psychotherapy (cognitive behavioural therapy and interpersonal therapy) had been offered without sufficient effect. Oral esketamine dose started at 0.5 mg/kg and was raised to 0.75 mg/kg. After two treatments, the dose was reduced to 0.5 mg/kg due to an unpleasant experience with difficulty swallowing and dyspnoea. Acute side effects were tingling sensations and numbness of the face, dizziness, muscle cramping and nausea. She reported increased intensity of both positive and negative emotions between the sessions. During her stay at the ward, someone was verbally aggressive to her. After this incident, the patient felt unsafe, and nightmares and flashbacks returned. The anxiety was exacerbated by the esketamine sessions, and the patient experienced an increase in suicidal ideation. After six weeks, the treatment was discontinued due to absence of mood improvement. The HDRS total score had increased from 22 at baseline to 26 in week six, whereas the IDS-SR had decreased from 58 to 32. The description of the patient experiences with esketamine treatment can be found in Table 1. She reported that the last esketamine session felt like a nightmare from the past, as if the trauma had taken over. The patient stabilized with clomipramine, fluvoxamine, and psychotherapy (EMDR and schema therapy) and was discharged from the ward after six months.

### 3.4 Case 4

A 34-year-old woman with TRD, PTSD and obsessive-compulsive and avoidant personality disorder presented with anxiety, low self-esteem, anhedonia, low mood, and suicidality. Her PTSD symptoms were a result of physical, mental and sexual abuse in her youth. Her coping strategies consisted of obsessive-compulsive behaviours and binge eating. She had a family history for MDD. Current pharmacotherapy consisted of imipramine and olanzapine, previous pharmacotherapy included SNRIs, TCAs, lithium, pregabalin, mirtazapine, antipsychotics and MAOIs. Electroconvulsive therapy (ECT) and schema therapy had been ineffective and severe anxiety prevented the patient from starting trauma-focussed therapy. Treatment with oral esketamine was titrated from 1.0 mg/kg to 1.5 mg/kg. After a few weeks, her mood improved and her anxiety, suicidal thoughts and obsessive behaviours subsided. HDRS and IDS-SR total scores decreased from 30 to 11 and 57 to 27 respectively. The patient noticed that the esketamine unlocked feelings of sadness and fear. She experienced that memories of traumatic experiences surfaced, but she was more able to tolerate the concomitant emotions. She was better able to look back on those situations rationally, without being overwhelmed by guilt: “I've noticed that at that time, I can better let go and observe, and put things into perspective much better”. The patient perspective can be found in Table 1. After 12 sessions of esketamine, she was discharged from the clinic and started trauma focussed psychotherapy.

### 3.5 Case 5

The fifth case is a 54-year-old female patient, diagnosed with severe, chronic TRD and complex PTSD. She suffered from depressed mood, suicidal ideations, loss of interest, inactivity, loss of energy, severe sleeping problems, nightmares, and anxiety. She experienced multiple traumas in the past, including sexual abuse and isolation on a psychiatric ward. Previous treatment for PTSD and TRD consisted of pharmacotherapy including SSRIs, TCAs, lithium, bupropion, MAOI and several benzodiazepines. Both ECT and intensive trauma therapy including EMDR did not lead to remittance of PTSD and TRD symptoms. Oral esketamine was administered with a starting dose of 0.5 mg/kg and titrated to 3 mg/kg. Initially her mood improved with a HDRS total score reduction of 10 points (from 25 to 15), IDS-SR scores were missing. The patient experienced intensive emotions after esketamine intake. She reported that her feelings seemed more accessible, and she started talking about her traumatic experiences. Trauma therapy was initiated and tolerated by the patient, feelings of helplessness reduced and behavioural changes were observed. Treatment has been ongoing for a year at this point. A process of acceptance emerged, and the PTSD Checklist (PCL-5) [36] score decreased with 9 points. During trauma therapy, previous positive effect on mood diminished and HDRS levels returned to a score of 25. A patient perspective was not requested in this case.

## 4 Discussion

This is the first report of repeated off-label treatment with generic oral esketamine in patients with TRD and comorbid PTSD. Prior extensive psycho- and pharmacotherapies had been unsuccessful in these patients, leading to severe suffering. Esketamine treatment duration ranged from six weeks to a year and was offered once or twice weekly. Depressive symptomatology was assessed with clinician rated (HDRS) and patient rated (IDS-SR) instruments. A decrease in HDRS was observed in four patients, a decrease in IDS-SR in three patients (available in four patients). In two cases, the change in HDRS and IDS-SR total scores showed a remarkable discrepancy. Previous trials evaluating clinician rated and patient reported outcomes in TRD patients have also described low correlations [37,38].

Clinical observations and patient's perspectives indicated that in four cases, esketamine treatment increased the patients' resilience, reduced the tendency to avoid exposure and increased their receptiveness to psychotherapy. One patient appeared to become more sensitive during esketamine treatment and experienced symptom worsening in response to a threatening situation. This highlights the need for a safe environment in which esketamine treatment should be offered. Common acute effects induced by esketamine were drowsiness, headache, dissociation, flashbacks, nightmares, and anxiety. These symptoms could be distressing for patients but were tolerated with emotional support from the staff. As experience in our clinic with this treatment for patients with TRD and PTSD developed, the staff became more capable of preparing patients, providing holding and guiding them through the process. We increasingly paid attention to set and setting which helped patients to surrender to the experience.

An important limitation of this study is the open label design. Because of the potential for bias and placebo effects, it is difficult to interpret the results. The increased resilience and receptiveness to psychotherapy might be due to other factors than the esketamine treatment, such as admission to the clinic, support from the staff or natural variation in symptoms over time. Furthermore, the positive effects on PTSD symptoms could be an indirect result through depressive symptom reduction.

The ability of ketamine augmentation to reinforce the effect of psychotherapy has been reported previously in PTSD and other mental disorders such as addiction, depression, and anxiety [28,30,32,33,39]. A recent review including RCTs investigating ketamine for PTSD describes prolonged effects and lower relapse rates if ketamine was combined with psychotherapy [25]. Ketamine is thought to facilitate the process of fear extinction, by promoting neuroplasticity and increasing synthesis of brain-derived neurotrophic factor (BDNF) [40]. This case series illustrates the potential of repeated oral esketamine administration in enabling trauma-focused therapy in a supportive setting for TRD and comorbid PTSD. This could be especially relevant for patients with PTSD, as psychological therapies show high dropout rates in this population [[41], [42], [43]]. However, this report also demonstrates the risks of enhanced sensitivity in these vulnerable patients. We therefore emphasize the importance of a safe setting and adequate support by the staff during (es)ketamine treatment.

## 5 Conclusions

We believe (es)ketamine administration within a psychotherapeutic framework holds promise for patients with severe and treatment resistant symptoms of depression and PTSD, in whom regular psychotherapy and pharmacotherapy is ineffective or not tolerated. Controlled trials with adequate sample sizes investigating the efficacy and optimal treatment methods (including dose, formulation, frequency, and number of administrations) of (es)ketamine are warranted, along with studies on the preferable timing and type of psychotherapy.

## Funding

None.

## Author contribution statement

J.K.E. Veraart: Conceived and designed the experiments; Performed the experiments; Analyzed and interpreted the data; Contributed reagents, materials, analysis tools or data; Wrote the paper.

M. van Westenbrugge; J.E. van Wulfften Palthe; A. van der Meij: Performed the experiments; Analyzed and interpreted the data; Contributed reagents, materials, analysis tools or data; Wrote the paper.

R.A. Schoevers: Conceived and designed the experiments; Analyzed and interpreted the data; Contributed reagents, materials, analysis tools or data; Wrote the paper.

J. de Jong: Analyzed and interpreted the data; Contributed reagents, materials, analysis tools or data; Wrote the paper.

## Data availability statement

Data included in article/supp. material/referenced in article.

## Declaration of competing interest

JV received a speakers fee from 10.13039/100008897Janssen Pharmaceuticals, outside the submitted work. RS received research funding for two randomized clinical trials with generic oral esketamine from the Netherlands Organisation for Health Research & Development and the 10.13039/501100010763National Health Care Institute, a speakers fee and investigator initiated research grant from 10.13039/100008897Janssen Pharmaceuticals, and consultancy fee from Clexio biosciences, both outside the submitted work. MvW, JvWP, AvdM and JdJ report no competing interests.
